# WaspBase: a genomic resource for the interactions among parasitic wasps, insect hosts and plants

**DOI:** 10.1093/database/bay081

**Published:** 2018-09-13

**Authors:** Longfei Chen, Kun Lang, Shoudong Bi, Jiapeng Luo, Feiling Liu, Xinhai Ye, Jiadan Xu, Kang He, Fei Li, Gongyin Ye, Xuexin Chen

**Affiliations:** 1 Ministry of Agriculture Key Lab of Molecular Biology of Crop Pathogens and Insects, Zhejiang University, 866 Yuhangtang Road, Hangzhou, China; 2 Anhui Agricultural University, 130 Changjiangxilu, Hefei, Anhui, China

## Abstract

Insect pests reduce yield and cause economic losses, which are major problems in agriculture. Parasitic wasps are the natural enemies of many agricultural pests and thus have been widely used as biological control agents. Plants, phytophagous insects and parasitic wasps form a tritrophic food chain. Understanding the interactions in this tritrophic system should be helpful for developing parasitic wasps for pest control and deciphering the mechanisms of parasitism. However, the genomic resources for this tritrophic system are not well organized. Here, we describe the WaspBase, a new database that contains 573 transcriptomes of 35 parasitic wasps and the genomes of 12 parasitic wasps, 5 insect hosts and 8 plants. In addition, we identified long non-coding RNA, untranslated regions and 25 widely studied gene families from the genome and transcriptome data of these species. WaspBase provides conventional web services such as Basic Local Alignment Search Tool, search and download, together with several widely used tools such as profile hidden Markov model, Multiple Alignment using Fast Fourier Transform, automated alignment trimming and JBrowse. We also present a collection of active researchers in the field of parasitic wasps, which should be useful for constructing scientific networks in this field.

## Introduction

Insects are the most widely distributed animal species on earth. Most insects are herbivores that cause huge yield losses when feeding on crops. Insects such as houseflies and mosquitos are vectors of pathogens that cause disease in humans and domesticated animals (1). To combat these insect pests, many methods have been developed, and some of which are used in agriculture. Insecticides are one of the main methods of pest control in agriculture. Unfortunately, overuse of insecticides causes serious environment pollution and food safety problems (2). Therefore, alternative, environment-friendly pest control methods should be developed.

Biological control is an environment-friendly pest control method. Parasitic wasps are well-known biological control agents (3, 4) as they are effective natural enemies of many economically important insect pests. Parasitic wasps are a group of hymenopteran insects that lay eggs in or on the bodies of hosts (5). The wasp larvae feed on the host until pupation and eventually kill the host (6). However, pest control using parasitic wasps has some apparent disadvantages such as wasp development lagging behind pest outbreaks and low-control efficiencies. Understanding the antagonistic interactions between parasitic wasps and their hosts is an important task to improve control efficiencies (7). At present, the genomes of 34 parasitic wasps have been deposited in public databases such as National Center for Biotechnology Information (NCBI). In addition, the genomes of six hosts of these wasps and eight plants that are damaged by these insect hosts are available. Among these species, five parasitic wasps (4,8–11), six insect hosts (12–18) and six plants (19–24) were publicly reported.

Though these data can be retrieved from NCBI, they are not well organized and thus have not been fully explored. Here, we collected the genome and transcriptome data of 34 parasitic wasps, 9 insect hosts and 8 plants from NCBI, i5k workspace@NAL (25) and InsectBase (7). Then, we constructed a database, which we named WaspBase, to serve as an integrated genomic resource for a tritrophic system of wasps, hosts and plants.

## Data resources

### Genomes

**Figure 1:**
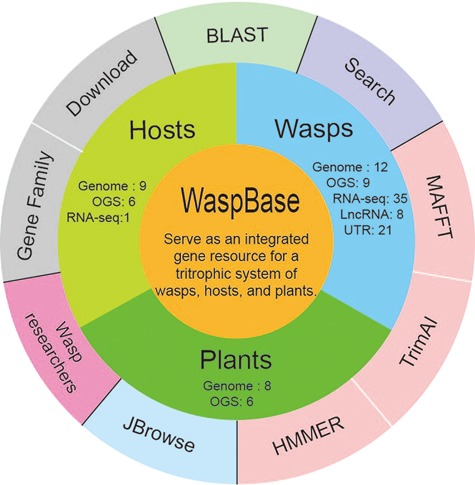
The design of WaspBase. The diagram shows the data and software used in WaspBase.

**Figure 2:**
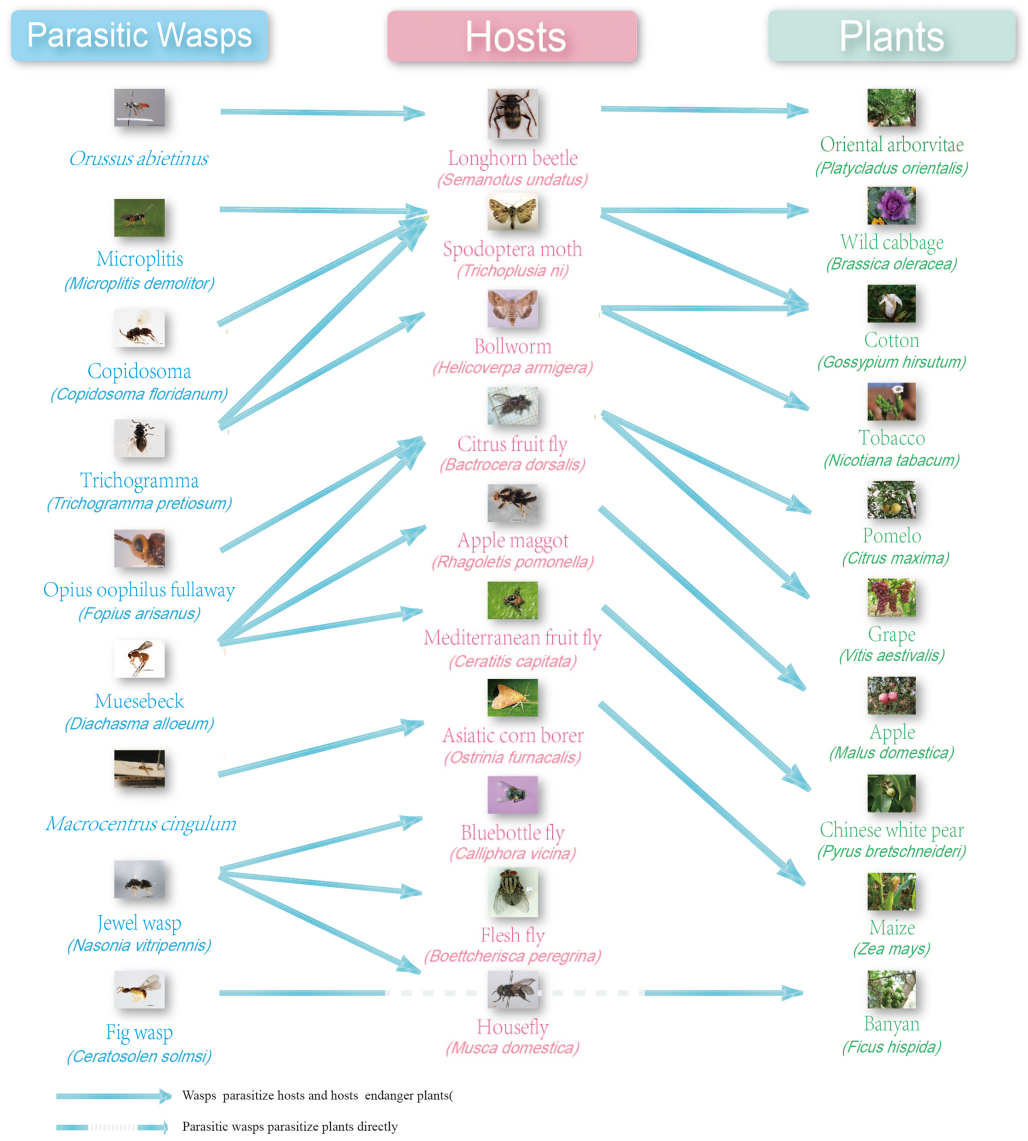
The parasitic wasps, hosts and plants included in the WaspBase. Dashed line: parasitic wasps parasitize plants but not insect hosts. Not dashed line: parasitic wasps parasitize the insect hosts or the insects damage plants.

We collected the genome data of 12 parasitic wasps from the NCBI including *Ceratosolen solmsi*, *Copidosoma floridanum*, *Cotesia vestalis*, *Diachasma alloeum*, *Fopius arisanus*, *Microplitis demolitor*, *Macrocentrus cingulum*, *Nasonia giraulti*, *Niphoparmena longicornis*, *Nasonia vitripennis*, *Orussus abietinus* and *Trichogramma pretiosum* (Figure 1) (8, 9, 11). The gene annotation files were obtained for nine parasitic wasps including *C. solmsi* (8), *C. floridanum* (10), *D. alloeum*, *F. arisanus* (4), *M. demolitor* (11), *M. cingulum*, *N. vitripennis* (9), *O. abietinus* and *T. pretiosum*. We then focused on these nine parasitic wasps with gene annotation information. There are nine insect hosts for these nine parasitic wasps, of which five have genome data and five have annotated genomes (12, 13). These five insect pests damage eight crops all of which have genome data, but six have annotation information (Figure 2). So, we collected a final genome data of nine parasitic wasps, five insect hosts and six plants (Table 1). The references reporting the interactions between parasitic wasps, insect hosts and plants were given in supplementary table S1.

**Table 1:** The genome data in the WaspBase

	**Species name**	**Accession ID**	**Source**
	*Ceratosolen solmsi*	GCF_000503995.1	NCBI
	*Copidosoma floridanum*	GCF_000648655.1	NCBI
	*Cotesia vestalis*	GCA_001675545.1	NCBI
	*Diachasma alloeum*	GCF_001412515.1	NCBI
	*Fopius arisanus*	GCF_000806365.1	NCBI
Wasps	*Macrocentrus cingulum*	-	InsectBase
	*Microplitis demolitor*	GCF_000572035.2	NCBI
	*Nasonia giraulti*	GCA_000004775.1	NCBI
	*Nasonia longicornis*	GCA_000004795.1	NCBI
	*Nasonia vitripennis*	GCF_000002325.3	NCBI
	*Orussus abietinus*	GCF_000612105.1	NCBI
	*Trichogramma pretiosum*	GCF_000599845.2	NCBI
	*Bactrocera dorsalis*	GCF_000789215.1	NCBI
	*Calliphora vicina*	GCA_001017275.1	NCBI
	*Ceratitis capitata*	GCF_000347755.2	NCBI
Hosts	*Helicoverpa armigera*	GCF_002156985.1	NCBI
	*Helicoverpa zea*	GCA_002150865.1	NCBI
	*Heliothis virescens*	GCA_002382865.1	NCBI
	*Manduca sexta*	-	InsectBase
	*Musca domestica*	GCF_000371365.1	NCBI
	*Brassica oleracea*	GCF_000695525.1	NCBI
	*Citrus maxima*	GCA_002006925.1	NCBI
	*Gossypium hirsutum*	GCF_000987745.1	NCBI
Plants	*Malus domestica*	GCF_000148765.1	NCBI
	*Nicotiana tabacum L*	GCF_000715135.1	NCBI
	*Pyrus x bretschneideri*	GCF_000315295.1	NCBI
	*Vitis aestivalis*	GCA_001562795.1	NCBI
	*Zea mays*	GCF_000005005.2	NCBI

### OGS

The General Feature Format version 3 (Gff3) files containing annotation information were downloaded with the genome data, and the official gene sets (OGSs) were extracted from the genome based on the annotation in the Gff3 file. Then, the nucleotide sequences and protein sequences of annotated genes were produced (Table 2).

**Table 2:** The protein and nucleotide dataset in the WaspBase

	**Species name**	**Accession ID**	**Source**
	*Ceratosolen solmsi*	GCF_000503995.1	NCBI
	*Copidosoma floridanum*	GCF_000648655.1	NCBI
	*Diachasma alloeum*	GCF_001412515.1	NCBI
	*Fopius arisanus*	GCF_000806365.1	NCBI
Wasps	*Macrocentrus cingulum*	-	InsectBase
	*Microplitis demolitor*	GCF_000572035.2	NCBI
	*Nasonia vitripennis*	GCF_000002325.3	NCBI
	*Orussus abietinus*	GCF_000612105.1	NCBI
	*Trichogramma pretiosum*	GCF_000599845.2	NCBI
	*Bactrocera dorsalis*	GCF_000789215.1	NCBI
	*Ceratitis capitata*	GCF_000347755.2	NCBI
Hosts	*Helicoverpa armigera*	GCF_002156985.1	NCBI
	*Manduca sexta*	-	InsectBase
	*Musca domestica*	GCF_000371365.1	NCBI
	*Brassica oleracea*	GCF_000695525.1	NCBI
	*Gossypium hirsutum*	GCF_000987745.1	NCBI
Plants	*Malus domestica*	GCF_000148765.1	NCBI
	*Nicotiana tabacum L*	GCF_000715135.1	NCBI
	*Pyrus x bretschneideri*	GCF_000315295.1	NCBI
	*Zea mays*	GCF_000005005.2	NCBI

### Transcriptomes

The raw data of 34 samples of parasitic wasps were downloaded from the NCBI SRA (Sequence Read Archive) data base (https://www.ncbi.nlm.nih.gov/sra). We assembled 22 transcriptomes using Trinity and TopHat-Cufflinks with default parameters (26, 27). Together with 21 other available transcriptomes, we collected a final transcriptome dataset of 573 RNA-Seq samples from 35 parasitic wasps (Table 3).

**Table 3:** The transcriptome data in the WaspBase

**Species name**	**Assembly**	**SRA**
*Aenasius bambawalei*	Trinity	SRR2966926
*Anastatus japonicus*	Trinity	SRR4034898
*Anisopteromalus calandrae*	Trinity	SRR2910690,SRR2910691
*Asobara tabida*	Not assembled	-
*Biorhiza pallida*	Trinity	ERR1353142,ERR1354102,ERR1354103,ERR1354104,ERR1354105, ERR1354106,ERR1354107,ERR1354108,ERR1354109,ERR1354110, ERR1354111,ERR1354112,ERR1354113,ERR1354114,ERR1354115, ERR1354116,ERR1354117,ERR1354118,ERR1354119,ERR1354354
*Ceratosolen solmsi*	TopHat-Cufflinks	SRR974922,SRR974923,SRR974924,SRR974925,SRR974926, SRR974927,SRR974928,SRR974929
*Copidosoma floridanum*	TopHat-Cufflinks	SRR1864696,SRR1864697
*Cotesia glomerata*	Not assembled	-
*Cotesia rubecula*	Not assembled	-
*Cotesia vestalis*	Not assembled	-
*Diachasma alloeum*	TopHat-Cufflinks	SRR2040481,SRR2041626
*Diachasmimorpha longicaudata*	Not assembled	SRR3336273,SRR3336336,SRR3336337
*Diadromus collaris*	Not assembled	SRR4294717,SRR1022346
*Fopius arisanus*	TopHat-Cufflinks	SRR1560649,SRR1560650,SRR1560651,SRR1560653
*Leptopilina boulardi*	Not assembled	ERR1109367,ERR1109368,ERR1109369,ERR1109370,ERR1109371, ERR1109372,ERR1109373,ERR1109374,ERR1109375,SRR559221,SRR559222
*Leptopilina clavipes*	Trinity	SRR921610
*Leptopilina heterotoma*	Trinity	SRR559223,SRR559224
*Lysiphlebus fabarum*	Not assembled	-
*Macrocentrus cingulum*	TopHat-Cufflinks	SRR2968845,SRR2968846
*Megastigmus spermotrophus*	Trinity	SRR1805073,SRR1805097,SRR1805105,SRR1805115
*Microctonus aethiopoides*	Not assembled	-
*Microplitis bicoloratus*	Not assembled	-
*Microplitis demolitor*	TopHat-Cufflinks	SRR955015,SRR955076,SRR955374,SRR955397
*Nasonia giraulti*	TopHat-Cufflinks	SRR3457435,SRR3457436,SRR3457437,SRR3457438,SRR3457439, SRR3457457,SRR1566028,SRR1566029,SRR1566030,SRR1566031, SRR1566032,SRR1566033,SRR1264518,SRR1264519,SRR1264521, SRR1264522,SRR1264523,SRR1264524,SRR1264525,SRR1264526, SRR1264527,SRR1264529,SRR1264530,SRR1264531
*Nasonia longicornis*	Not assembled	-
*Nasonia vitripennis*	Not assembled	-
*Orussus abietinus*	TopHat-Cufflinks	ERR1333211,SRR1850925,SRR1850924,SRR921626
*Ostrinia furnacalis*	Trinity	DRR018822,DRR018823,DRR018824,DRR018825,DRR018826, DRR018827,DRR030133,DRR030134,DRR030135,DRR030136, DRR030137,DRR030138,DRR030139,DRR030140,DRR030141, DRR030142,SRR1032037,SRR1032038,SRR1226611,SRR1265986, SRR1560699,SRR1560709,SRR1560711,SRR1565323,SRR1640337, SRR1640339,SRR1640341,SRR3189772,SRR3204354,SRR3204356, SRR3204357,SRR3374123,SRR3374124,SRR3374125
*Psyttalia concolor*	Trinity	SRR1593901,SRR1593902
*Psyttalia lounsburyi*	Trinity	SRR1593906,SRR1593907,SRR1593908
*Pteromalus puparum*	Not assembled	-
*Spalangia endius*	Trinity	SRR2954670,SRR2954673,SRR2954678,SRR2954681,SRR2954683, SRR2954686,SRR2954688,SRR2954692,SRR2954704,SRR2954706, SRR2954708,SRR2954710,SRR1038395
*Telenomus podisi*	Trinity	SRR1274857,SRR1274858
*Trichogramma chilonis*	Trinity	SRR3756972,SRR3756974,SRR3756975,SRR3756979
*Trichogramma pretiosum*	TopHat-Cufflinks	SRR1826957,SRR1826958
*Venturia canescens*	Trinity	ERR791800

### lncRNA

Long non-coding RNAs (lncRNAs) are transcribed RNA molecules >200 nucleotides in length that are not protein coding (28, 29). We predicted lncRNAs of eight parasitic wasps using a previously reported pipeline (30). In total, we predicted 49 607 lncRNAs from eight parasitic wasps.

### UTR

We developed a pipeline to predict untranslated regions (UTR) from the transcriptomes and genomes using TransDecoder-V5.3.0 (https://github.com/TransDecoder/TransDecoder), identifying the UTR sequences of 21 parasitic wasps.

### Gene families

**Figure 3:**
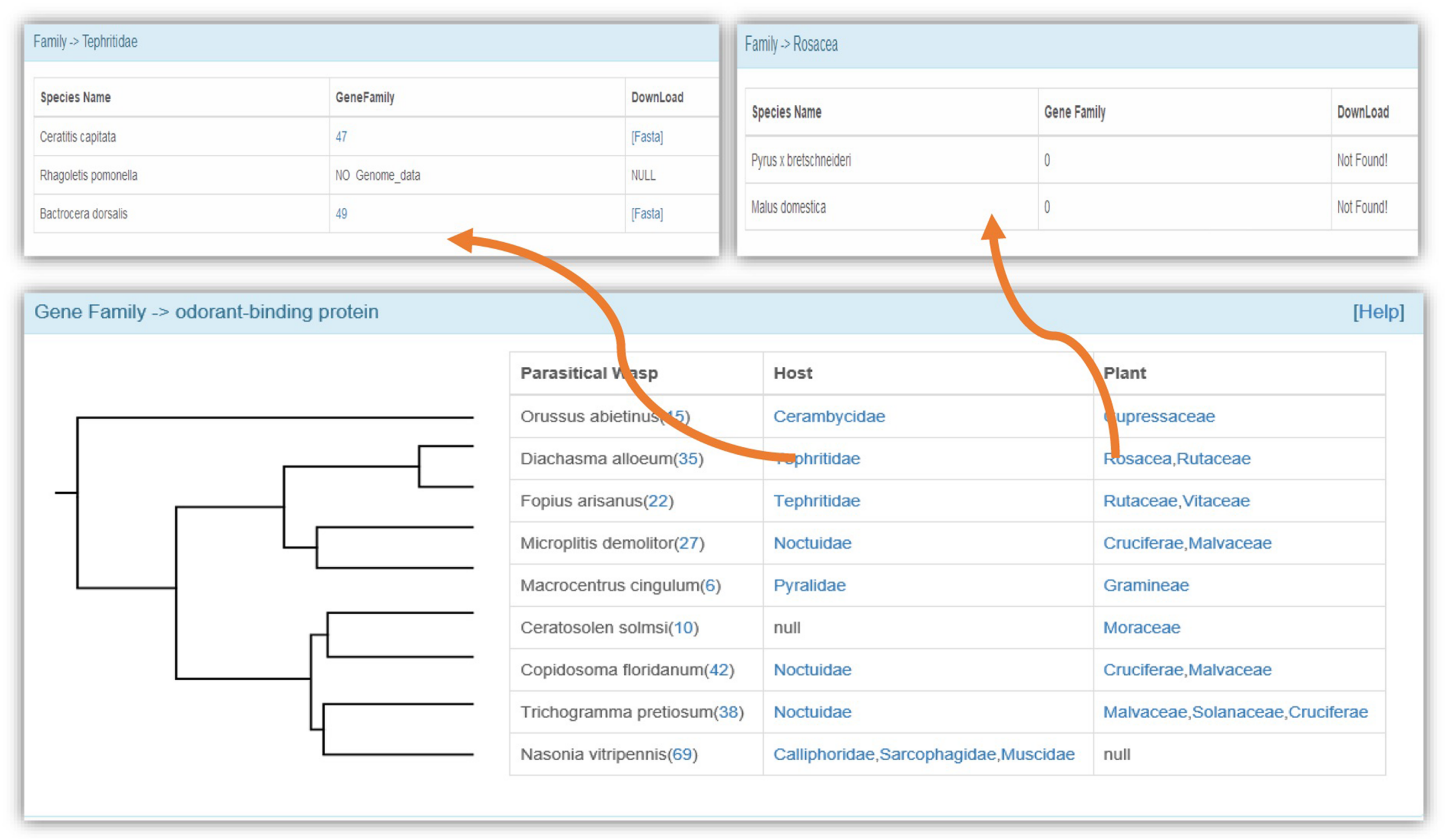
The identified gene families in the WaspBase.

We used manual annotation by Blastp against known genes (e-value = 10^−5^), GO annotation and phylogenetic analysis to identify the members of a gene family. We obtained the information of 25 gene families that have been widely studied, including those related to chemoreception, the immune system and detoxification (Figure 3). We also provided a web server for phylogenetic analysis of selected gene members, and we use ClustalW2 (31) to construct a phylogenetic tree by the neighbor-joining clustering method. The bootstrap value was set as 500. The Newick Utilities V1.6 (32) was used to display the phylogenetic tree.

## Database construction

### Database system implementation

WaspBase was developed on an Apache HTTP (Apache 2.4.25) server in a Linux (RedHat 4.8.2) operating system. The web pages were written using PHP (PHP 5.6.30), html language, Cascading Style Sheets and JavaScript. All data are stored in the MySQL (MySQL 5.7.17) environment. The Apache server handles queries from web clients through PHP scripts to perform searches.

### Search function

WaspBase provides search function using keywords, gene ID, gene names, annotation keywords, KEGG ID, KEGG annotation (33), PFam ID or Pfam annotation (34). Once a gene is searched for, all related gene information was presented in the result webpages. The genes from parasitic wasps, insect hosts and plants were given in the searched results.

### Tools module

The tools module contains Basic Local Alignment Search Tool (BLAST) (35), profile hidden Markov model (HMMER), Multiple Alignment using Fast Fourier Transform (MAFFT), automated alignment trimming (TrimAl) and JBrowse (36).

BLAST (35) is provided using the Web-based BLAST server 2.6.0+. The data used for nucleotide BLAST (BLASTN, TBLASTN) searches include 12 insect genomes and 9 insect OGSs. The protein data used for amino acid BLAST (BLASTP, TBLASTX, BLASTX) searches contain nine insect protein sequences. In the BLAST results webpage, users can choose to display top 5 hits, top 10 hits or all hits. The top five BLAST hits are used as default. User can also adjust other parameters such as similarity percentage and BLAST score. Links of the BLAST hits were given to directly connect to NCBI for full annotation information. All sequence can be downloaded.

**Figure 4:**
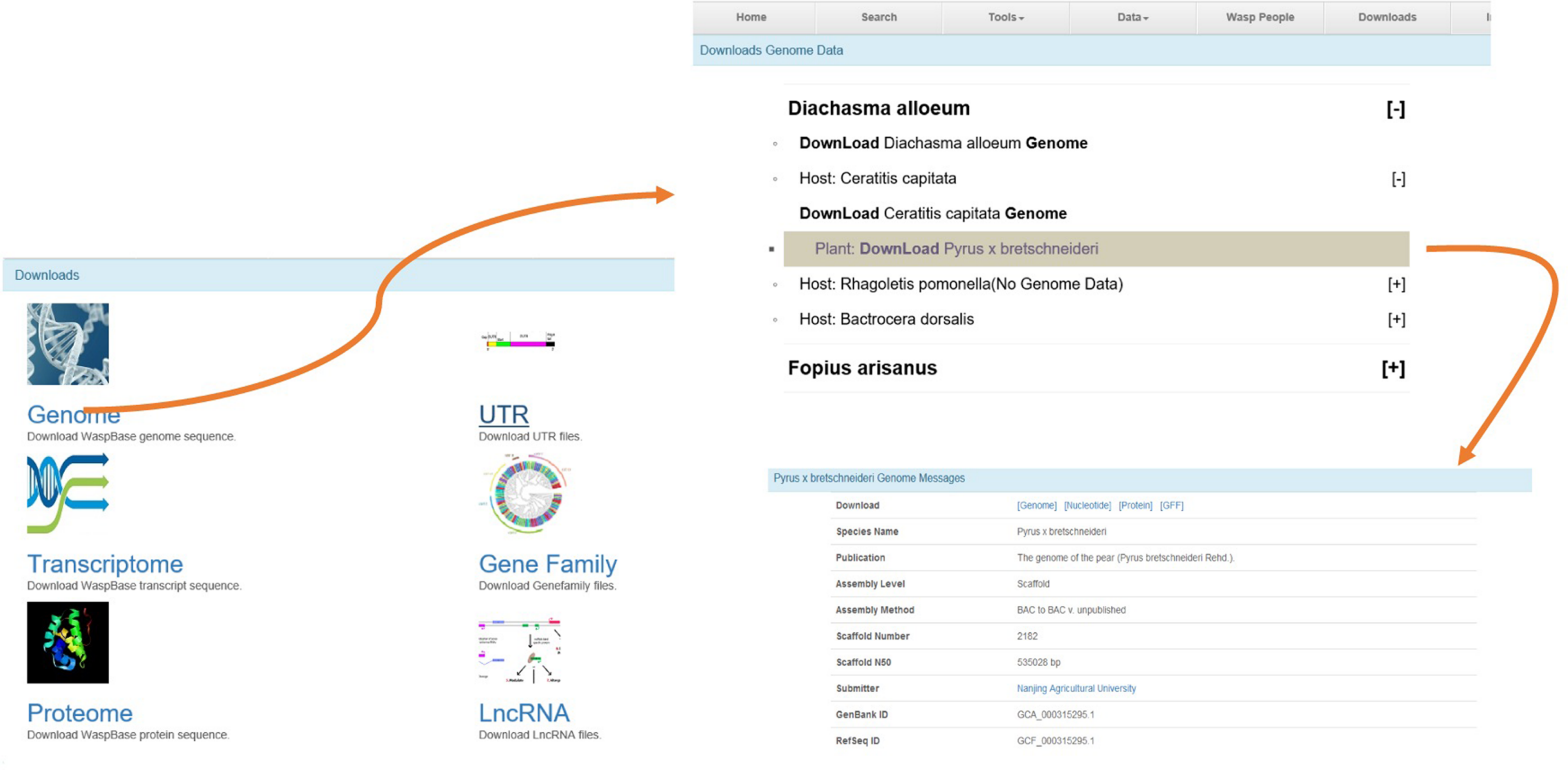
The Download page of WaspBase. The genomes, transcriptomes and OGSs of parasitic wasps and insect hosts are provided together for the convenience of download.

Multiple sequence alignment (MSA) is important for evolutionary analyses. MAFFT (37) is a widely used program for MSA analysis because of its high performance. WaspBase provides a web server of MAFFT and uses TrimAl to trim the aligned sequences (38). To use MAFFT web server, users need to input the sequences in FASTA format with either the default parameters or the customized parameters. To use TrimAl, users need to input the aligned sequences at the TrimAl webpage. The trimmed sequences are showed at TrimAl result webpage. If the number of sequence is more than four, a phylogenetic tree can be constructed using the abovementioned method.

A web server of HMMER is provided to search sequence homologs and to make sequence alignments. It uses probabilistic models called profile hidden Markov models (profile HMMs) (39). To use HMMER, users input the protein sequences at the HMMER webpage. After running the HMMER, the protein sequences are used to search against the Pfam database and the results of protein domain information will be showed at the HMMER result webpage.

### Genome visualization

JBrowse is a well-known browser that displays genome annotations by integrating the databases and interactive web pages (36). We used JBrowse in WaspBase to provide interactive views of annotations along with the genome scaffolds. The genome data and the Gff3 files required for JBrowse are stored in a MySQL database using prepare-refseqs.pl, flatfile-to-json.pl, add-bam-track.pl and add-track-json.pl provided by BioPerl. In WaspBase, JBrowse visualizes the annotations and transcriptomes as tracks on the browser for Coding Sequence and coverage of the transcriptome reads. Pop-up balloons in the gene model track display links to gene sequences of interest.

### Wasp researchers

To construct a scientific network in the field of parasitic wasp research, we performed reference mining of parasitic wasp studies, which yielded 189 references. Based on publications in the last 5 years, we collected a list of active researchers studying parasitic wasps.

### Download

All data can be downloaded, including genomes, transcriptomes, UTR, Gene families and lncRNA. For the convenience of downloading, the gene data of parasitic wasps, insect pests and plants are provided for download at the same webpage (Figure 4).

## Conclusions

We constructed WaspBase for parasitic wasps and their corresponding insect hosts and plants. WaspBase provides conventional functions of search, download, domain analysis and phylogenetic analysis, JBrowse display of annotations and other functions described herein. In addition to genomes and transcriptomes, WaspBase also provides lncRNA, UTR and gene family information. A typical feature of WaspBase is that we integrated the gene information of parasitic wasps, their insect hosts and plants targeted by insect pests. Thus, gene data of the tritrophic system in food chains (parasitic wasp–insect pest–plant) were analyzed together, which should be useful for studying cross-species regulation in parasitism and convergent evolution analysis among wasps, hosts and plants.

### Future plan

1. As the cost of sequencing has been significantly reduced in recent years, the genomes of an increasing number of parasitic wasps will be sequenced. We plan to update WaspBase periodically to keep the database up-to-date.
2. Genome annotation is still a time-consuming task and significantly lags behind genome sequencing. We noticed that a number of parasitic wasp genomes are not annotated at present though their genome sequences have been uploaded in the NCBI genome database. We will annotate these genomes using OMIGA (Optimized Maker-Based Insect Genome Annotation) (40), a genome annotation pipeline that we developed.
3. It is important to understand cross-species regulation mechanisms and convergent evolution in parasitism. To this end, we will carry out a systematic analysis of more gene families from the OGSs of ‘wasps–insects–plants’, which should be useful to improve control efficiencies in biological control.

## Supplementary Material

Supplementary Data

## Notes

**Database URL:** http://www.insect-genome.com/waspbase/.
