# Supplementary material for: WaspBase: a genomic resource for the interactions among parasitic wasps, insect hosts and plants
Source: Database (Oxford). 2018 Sep 13;2018:bay081. doi: 10.1093/database/bay081 (PMC6146128; doi:10.1093/database/bay081)
Supplement: Supplementary Data [file bay081_supp.docx]

**Table S1** **The references of published genomes of parasitic wasp, insect hosts and plants**

| **Type** | **Species Name** | **Species Name** | **Article Title** | **Journal** | **Year** |
| --- | --- | --- | --- | --- | --- |
| **Wasp-Host** | *Copidosoma floridanum* | *Trichoplusia ni* | Bt cotton producing Cry1Ac and Cry2Ab does not harm two parasitoids, *Cotesia marginiventris* and *Copidosoma floridanum.* | *Scientific Reports* | 2018 |
|  | *Diachasma alloeum* | *Rhagoletis pomonella* | Behavioral evidence for host fidelity among populations of the parasitic wasp, *Diachasma alloeum* (Muesebeck) | *Naturwissenschaften* | 2005 |
|  | *Fopius arisanus* | *Bactrocera dorsalis* | Regional Suppression of Bactrocera Fruit Flies (Diptera: Tephritidae) in the Pacific through Biological Control and Prospects for Future Introductions into Other Areas of the World | *Insects* | 2012 |
|  | *Macrocentrus cingulum* | *Ostrinia furnacalis* | The genomic features of parasitism, Polyembryony and immune evasion in the endoparasitic wasp *Macrocentrus cingulum* | *BMC Genomics* | 2018 |
|  | *Microplitis demolitor* | *Trichoplusia ni* | Permissiveness of lepidopteran hosts is linked to differential expression of bracovirus genes. | *Virology* | 2016 |
|  | *Nasonia vitripennis* | *Calliphora vicina* | Depth and type of substrate influence the ability of *Nasonia vitripennis* to locate a host | *Journal of Insect Science* | 2013 |
|  | *Nasonia vitripennis* | *Boettcherisca peregrina* | Effects of host (*Boettcherisca peregrina*) copper exposure on development, reproduction and vitellogenesis of the ectoparasitic wasp, *Nasonia vitripennis* | *Insect Science* | 2009 |
|  | *Nasonia vitripennis* | *Musca domestica* | Identification of a small pacifastin protease inhibitor from *Nasonia vitripennis* venom that inhibits humoral immunity of host (*Musca domestica*) | *Toxicon* | 2017 |
|  | *Orussus abietinus* | *Semanotus undatus* | *Orussus abietinus* scop (Hym, Orussoidea) as a parasite of *Semanotus undatus* L larvae (Col, Cerambycidae) | *Anzeiger Für Schädlingskunde Pflanzenschutz Umweltschutz* | 1984 |
|  | *Trichogramma pretiosum* | *Manduca sexta* | Effect of Mass Releases of *Trichogramma pretiosum* Against Lepidopterous Pests on Processing Tomatoes in Southern California, with Notes on Host Egg Population Trends | *Journal of Economic Entomology* | 1978 |
|  | *Trichogramma pretiosum* | *Trichoplusia ni* | Temperature Effects on the Development and Reproduction of Three Trichogramma (Hymenoptera: Trichogrammatidae) Species Reared on *Trichoplusia ni* (Lepidoptera: Noctuidae) Eggs | *Journal of Insect Science* | 2015 |
|  | *Trichogramma pretiosum* | *Helicoverpa armigera* | Thermal Requirements and Performance of the Parasitoid *Trichogramma pretiosum* (Hymenoptera: Trichogrammatidae) on *Helicoverpa armigera* (Lepidoptera: Noctuidae) Eggs Under Variable Temperatures. | *Environmental Entomology* | 2017 |
| **Host-Plant** | *Helicoverpa armigera* | *Gossypium hirutum* | Dynamic transcriptome analysis and volatile profiling of *Gossypium hirsutum* in response to the cotton bollworm *Helicoverpa armigera* | *Scientific Reports* | 2015 |
|  | *Helicoverpa armigera* | *Nicotiana tabacum L* | Larval feeding induced defensive responses in tobacco: comparison of two sibling species of Helicoverpa with different diet breadths | *Planta* | 2007 |
|  | *Ostrinia furnacalis* | *Zea mays* | The Asian corn borer *Ostrinia furnacalis* feeding increases the direct and indirect defence of mid-whorl stage commercial maize in the field. | *Plant Biotechnology Journal* | 2018 |
|  | *Rhagoletis pomonella* | *Malus domestica* | Ammonium carbonate is more attractive than apple and hawthorn fruit volatile lures to *Rhagoletis pomonella* (Diptera: Tephritidae) in Washington State | *Environmental Entomology* | 2014 |
|  | *Trichoplusia ni* | *Brassica oleracea* | Plant growth regulator-mediated anti-herbivore responses of cabbage (*Brassica oleracea*) against cabbage looper *Trichoplusia ni* Hübner (Lepidoptera: Noctuidae) | *Pesticide Biochemistry & Physiology* | 2016 |
|  | *Trichoplusia ni* | *Gossypium hirutum* | Effects of host plant, *Gossypium hirsutum* L., on sexual attraction of cabbage looper moths, *Trichoplusia ni* (Hübner) (Lepidoptera: Noctuidae) | *Journal of Chemical Ecology* | 1994 |
| **Wasp-Plant** | *Ceratosolen solmsi* | *Ficus hispida* | Obligate mutualism within a host drives the extreme specialization of a fig wasp genome | *Genome Biology* | 2013 |
